# Supplementary material for: Heat stress disrupts early development and photosymbiosis in Cassiopea jellyfish
Source: PLoS One. 2025 Nov 12;20(11):e0323922. doi: 10.1371/journal.pone.0323922 (PMC12611107; doi:10.1371/journal.pone.0323922)
Supplement: Attachment — Submitted filename: Revision PlosOne.docx [file pone.0323922.s008.docx]

The manuscript PONE-D-25-20894 titled: Heat stress disrupts early development and photosymbiosis in *Cassiopea* jellyfish has been revised. While the topic is relevant and of broad scientific interest, the presentation of the findings lacks sufficient depth, and a more cautious interpretation of the results is recommended, supported by a clearer and more comprehensive presentation of the data. For these reasons I recommend a major revision.

**Major** **issues**

The experimental design is not well presented. You should state how many polyps (pseudo replicates) were in each small well (replicates). You should also better clarify how water changes were performed and how optimal living conditions were maintained throughout the experiment. I recommend including a paragraph in the Discussion section addressing the limitations of this study and outlining how improving the design.

The presentation of the results could be significantly improved. While I do not recall the specific formatting guidelines of Plos One, I strongly recommend separating the Results and Discussion sections to enhance clarity and readability. In the Result section, you should provide numerical values and standard deviation of each obtained result.

The chosen temperature value (34 °C) may be extreme to conduct your experiment. You should consider the possibility that also aposymbionts could show inverted ephyrae due to heat stress only and not due to an impairment of the symbiosis. This possibility should be addressed when interpreting the results and discussing potential confounding factors. It would be beneficial to include a discussion on the natural environmental conditions experienced by the polyps. What potential stressors do they encounter in the wild, and how might these compare to the experimental conditions?

Minor issues were listed below.

**Introduction**

Line 53. Change in ‘develop into new polyps’

In general, check the word ‘metamorphosis’. You should use it to indicate the formation of a new polyp from the larva/polyp bud. The formation of the ephyra stage is the ‘strobilation’.

Line 68: specify: in Scleractinians instead of in corals.

Line 156. About the sentence: ‘For example, *Aurelia aurita* strobilates after prolonged cold periods (19), whereas species like *Cephea* 158 *cephea* and *Rhopilema verrilli* respond to elevated temperatures (20,21)’, I suggest considering also papers on strobilation of wild polyps.

Line 158. About the sentence: ‘Unlike these species, *Cassiopea* relies primarily on colonization by endosymbiotic *Symbiodinium* to initiate strobilation’, what does it means? Is the strobilation continuous? You should explain in detail this crucial point by summarizing results of the cited papers.

**Methods**

Line 138: define ‘inverted ephyrae’

Line 154: prevented?

I recommend adding a table to summarize objectives, research questions for each objective, used methods for each research question.

**Results**

Line 161: please, check grammar of this sentence

Line 196: change in ‘chlorophyll *a*’
