# Supplementary material for: Heat stress disrupts early development and photosymbiosis in Cassiopea jellyfish
Source: PLoS One. 2025 Nov 12;20(11):e0323922. doi: 10.1371/journal.pone.0323922 (PMC12611107; doi:10.1371/journal.pone.0323922)
Supplement: Attachment — Submitted filename: 20250816_JellyFish_Rebuttal.docx [file pone.0323922.s010.docx]

The manuscript PONE-D-25-20894 titled: Heat stress disrupts early development and photosymbiosis in Cassiopea jellyfish has been revised. While the topic is relevant and of broad scientific interest, the presentation of the findings lacks sufficient depth, and a more cautious interpretation of the results is recommended, supported by a clearer and more comprehensive presentation of the data. For these reasons I recommend a major revision.

**Response:** We thank the reviewers for their constructive feedback. In response, we clarified the experimental design, included numerical values and standard deviations where appropriate, added a new section on study limitations and future directions, expanded the discussion of natural environmental conditions. We believe these changes have strengthened the manuscript.

**Major issues**

1. The experimental design is not well presented. You should state how many polyps (pseudo replicates) were in each small well (replicates). You should also better clarify how water changes were performed and how optimal living conditions were maintained throughout the experiment.

**Response:** We appreciate the reviewer’s thoughtful feedback on the clarity of the replicates and maintenance of the 6-well plates. In response, we have clarified the number of polyps per well, rather than just stating the total number of polyps in the entire plate. While there is still some uncertainty, due to there being either 12 or 13 polyps per well, we believe this to be much clearer than simply stating 50 polyps overall. We also added a more detailed account for how the water changes were performed after infection. Finally, we added information regarding the incubators that held constant light and temperature for the culturing and growth of the symbionts and polyps throughout the experiment. Specifically, we clarified the use of two separate incubators to simulate the two different temperatures used in the experiment.

2. I recommend including a paragraph in the Discussion section addressing the limitations of this study and outlining how improving the design.

**Response:** We appreciate the reviewer’s suggestion. We have added a new “Limitations and Future Directions” section at the end of the Discussion.

Revised text: As an initial exploration into how heat stress impacts *C. xamachana*, our study reveals elevated temperatures impacts early development. At the same time, we recognize several limitations that offer opportunities for future research. Although our observations highlight disruptions to strobilation, assessing the underlying molecular mechanisms that drive these responses was beyond the scope of this study. Integrating transcriptomic or metabolomic approaches in future work could help identify key regulatory pathways or symbiont-derived cues altered by thermal stress. Moreover, our design focused on two discrete temperature conditions. Expanding the study to include a broader and more ecologically relevant temperature gradient, as well as varying exposure durations, would help define thresholds and the temporal progression of stress effects with greater ecological significance. Finally, *C. xamachana* polyps do not undergo strobilation without symbiont colonization. This currently prevents the use of heat-stressed aposymbiotic controls. However, future studies that identify the biochemical triggers of strobilation may enable such experimental manipulations, allowing researchers to disentangle host responses from those of the symbionts.

3. The presentation of the results could be significantly improved. While I do not recall the specific formatting guidelines of Plos One, I strongly recommend separating the Results and Discussion sections to enhance clarity and readability.

**Response:** We appreciate the reviewer’s feedback on improving clarity. While PLOS ONE permits both combined and separate formats, we have opted to retain a combined Results and Discussion section because our findings are straightforward and organized by distinct topics: the effects of heat stress on host strobilation, host development abnormalities, and host reproduction. We believe that presenting and interpreting each topic together improves readability and allows for a more immediate understanding of the implications of each result.

In response to the reviewer’s concern, we have revised the section to ensure that the presentation of results is clearer and more distinct within each thematic subsection. Specifically, we use the first paragraph of each section to summarize all relevant results. We hope this revised structure achieves better clarity while preserving the contextual flow of the discussion.

4. In the Result section, you should provide numerical values and standard deviation of each obtained result.

**Response:** We have revised the results to add numerical values wherever possible. Numerical values were added in regard to cultured algal density, chlorophyll concentration, ephyra algal cell density, and polyp asexual budding quantifications. Standard deviations were added for polyp asexual budding quantifications and ephyra algal cell density. Standard deviation was not added for chlorophyll concentrations because there were so few measurements taken, so we were able to report them all.

5. The chosen temperature value (34 °C) may be extreme to conduct your experiment. You should consider the possibility that also aposymbionts could show inverted ephyrae due to heat stress only and not due to an impairment of the symbiosis. This possibility should be addressed when interpreting the results and discussing potential confounding factors. It would be beneficial to include a discussion on the natural environmental conditions experienced by the polyps. What potential stressors do they encounter in the wild, and how might these compare to the experimental conditions?

**Response:** We appreciate the reviewer’s thoughtful comment and agree that 34 °C represents a high thermal condition. This temperature was chosen to simulate an extreme heat stress scenario, such as those increasingly observed during marine heatwaves. While this temperature may exceed average environmental conditions, it remains within the range of short-term maxima recorded in shallow coastal habitats, where polyps naturally occur (Fitt and Costley, 1998, Journal of Experimental Marine Biology and Ecology, Fitt et al., 2025, Oceans).

We fully acknowledge the possibility that the inverted ephyrae observed in the aposymbiotic treatment could result from heat stress alone, rather than solely from the disruption of symbiosis. We have now addressed this potential confounding factor in the revised Results and Discussion section by creating a new subsetction ‘*Potential environmental factors impacting Cassiopea life-history’*. Furthermore, we have expanded the discussion to include a consideration of natural environmental conditions experienced by polyps *in situ*. While 34°C is higher than average seasonal temperatures, episodic events such as heatwaves can lead to transient temperature spikes exceeding this threshold. We also suggested that future studies explore long-term exposure to 34°C temperature, and examine combined stressor effects to disentangle the specific impacts of thermal stress versus symbiosis disruption.

**Minor issues**

**Introduction**

7. Line 53. Change in ‘develop into new polyps’

Revised.

8. In general, check the word ‘metamorphosis’. You should use it to indicate the formation of a new polyp from the larva/polyp bud. The formation of the ephyra stage is the ‘strobilation’.

We have carefully reviewed the manuscript and corrected the usage of metamorphosis and strobilation.

9. Line 68: specify: in Scleractinians instead of in corals.

Revised.

10. Line 156. About the sentence: ‘For example, Aurelia aurita strobilates after prolonged cold periods (19), whereas species like Cephea 158 cephea and Rhopilema verrilli respond to elevated temperatures (20,21)’, I suggest considering also papers on strobilation of wild polyps.

**Response:** We agree that understanding strobilation in natural contexts is important. However, due to the challenges of pinpointing environmental triggers *in situ*, most studies rely on polyps collected from the wild and tested under controlled laboratory conditions. Some studies infer natural triggers through correlations between field observations and environmental parameters, often complemented by lab experiments. In response, we have now cited two relevant studies:

Omori (1995), which monitored seasonal temperature changes in Tokyo Bay and demonstrated that declining temperatures likely trigger strobilation in *Aurelia aurita*.

Purcell et al. (2009), which analyzed multi-year field data and found that strobilation in *Aurelia labiata* was associated with higher temperature and high light conditions in natural settings.

These studies illustrate how natural strobilation cues are inferred through correlative approaches. We have revised the manuscript to incorporate these references.

Revised text: Strobilation cues in jellyfish vary widely among species and can be influenced by environmental factors such as temperature, salinity, and light exposure. These patterns are typically inferred by correlating environmental variables with observed strobilation events and are often validated through laboratory studies. For example, *Aurelia aurita* strobilates after prolonged cold periods (19, Omori 1995), whereas species like *Cephea cephea* and *Rhopilema verrilli* respond to elevated temperatures (20, 21). In *Aurelia labiata*, strobilation has been associated with high-temperature and high-light conditions in natural settings (Purcell et al. 2009).

Added references:

Omori, M., Ishii, H. and Fujinaga, A.I., 1995. Life history strategy of Aurelia aurita (Cnidaria, Scyphomedusae) and its impact on the zooplankton community of Tokyo Bay. ICES Journal of Marine Science, 52(3-4), pp.597-603.

Purcell, J.E., Hoover, R.A. and Schwarck, N.T., 2009. Interannual variation of strobilation by the scyphozoan Aurelia labiata in relation to polyp density, temperature, salinity, and light conditions in situ. Marine Ecology Progress Series, 375, pp.139-149.

11. Line 158. About the sentence: ‘Unlike these species, Cassiopea relies primarily on colonization by endosymbiotic Symbiodinium to initiate strobilation’, what does it means? Is the strobilation continuous? You should explain in detail this crucial point by summarizing results of the cited papers.

We thank the reviewer for helping us to further clarify a key point when presenting the background information regarding the life history of our studied species *Cassiopea xamachana.* We have added that information in the same section (Line 198).

Revised text: In *Cassiopea xamachana*, however, this transition is dependent on successful colonization by endosymbiotic Symbiodinium spp., which acts as an essential developmental driver (Hofmann et al., 1978; Colley and Trench, 1985). The presence of these dinoflagellates appears to be a critical environmental and physiological cue required to trigger the transition from the polyp stage (scyphistoma) to the strobila stage, ultimately leading to the release of juvenile medusae (ephyrae). In brief, the transition is induced when symbionts are ingested and phagocytosed by gastrodermal cells, migrating basally within three days (Colley and Trench, 1985; Fitt and Trench, 1983). By day eight, symbiont-containing host cells relocate to the mesoglea and proliferate (Colley and Trench, 1985). Strobilation occurs around three weeks post-colonization, influenced by symbiont species, temperature, and nutrition (Fitt and Costley, 1998).

**Methods**

12. Line 138: define ‘inverted ephyrae’

We referred as an inverted bell ephyra, based on the morphology observations made in Thayer et al., 2022 “Severe seawater acidification causes a significant reduction in pulse rate, bell diameter, and acute deterioration in feeding apparatus in the scyphozoan medusa Cassiopeia sp.” Invertebr Zool (See figure 5F). We have referenced this paper in the Introduction and changed the term inverted ephyra to inverted bell ephyra throughout the text.

13. Line 154: prevented?

Revised.

14. I recommend adding a table to summarize objectives, research questions for each objective, used methods for each research question.

**Response:** While we recognize the value of summarizing objectives, research questions, and methods in a table format, we have opted to retain this information in the narrative for clarity and flow. Because the study follows a relatively straightforward rational and method section, we felt that integrating this information into the text maintains cohesion and avoids redundancy. We have revised the Methods sections to make alignment between objectives, questions, and methods more explicit and easier to follow. Specifically, we have reorganized the Methods section so that the order of measurement descriptions now matches the sequence in which the results are presented.

We have also revised the Introduction to clearly state the focus of this study:

Revised text: A combination of morphological observations, symbiont density quantification, and chlorophyll concentration analysis was used to quantitatively and qualitatively assess the effects of heat stress on *C. xamachana* strobilation timing, ephyrae development and asexual reproduction patterns.

**Results**

15. Line 161: please, check grammar of this sentence

Revised.

16. Line 196: change in ‘chlorophyll a’

Revised.
