# Supplementary material for: Heat stress disrupts early development and photosymbiosis in Cassiopea jellyfish
Source: PLoS One. 2025 Nov 12;20(11):e0323922. doi: 10.1371/journal.pone.0323922 (PMC12611107; doi:10.1371/journal.pone.0323922)
Supplement: Attachment — Submitted filename: 20251027_JellyFish_Rebuttal.docx [file pone.0323922.s011.docx]

Celeste Robinson and colleagues present the manuscript ‘Heat stress disrupts early development and photosymbiosis in Cassiopea jellyfish’. The authors performed an interesting experiment in which they heat-stressed algal symbionts native to *Cassiopea xamachana*, and subsequently used the stressed symbionts for the inoculation of aposymbiotic polyps of the jellyfish. In the two heat-stressed group, the authors compare the effects of continuous heat stress (where heat stress is maintained after inoculation of polyps) vs. a recovery phase (where control temperatures are resumed after inoculation), and find interesting stress responses with regard to strobilation in control jellyfish.

While the revision has made some important changes which have strengthened the paper, there are still a few issues that need addressing. I hope my comments below help shape a revision of this interesting work.

**Response:** We thank the reviewer for their constructive feedback. In response, we clarified methodological details, corrected normalization of algal counts, and added missing figures and standard deviations where available. We also expanded the discussion on the metabolic role of photosymbiosis and its link to strobilation, integrating additional literature. We believe these changes have strengthened the manuscript.

**Main concerns:**

1) The main issue is probably that while the authors show interesting results and patterns, the data are not always robust. For instance, the authors decided not to add standard deviations/errors to chlorophyll a content plots due to having only ‘few measurements’. While the affected results make sense biologically, replication would have been necessary to make this result more robust. This limitation should at least be mentioned in the discussion, and the interpretation should be toned down accordingly – as already mentioned by one of the previous reviewers. I do not feel this point has been sufficiently addressed yet.

**Response:** We thank the reviewer. We started with a large volume of algal culture, and each time we extracted a sample, it introduced a chance of contamination. To limit this exposure to our stock culture, we chose to take fewer samples. To supplement the dataset, we included representative images (Figure 4c). We acknowledge that additional replicates would strengthen the statistical robustness of the results, and we have accordingly moderated our conclusions in the discussion section to reflect this limitation.

2) The discussion is overall well written, but I don’t find it justified that there is not really any attention paid to the potential metabolic role of photosymbiosis in strobilation – it is brushed over very briefly in one sentence, but not really discussed. Considering the importance of symbiotic nutrient cycling in the adult medusa (and many other photosymbiotic Cnidaria), I strongly feel potential host starvation and energy limitation should be discussed in the context of failed strobilation. I understand that the authors are more interested in immunity, but immunity and nutrient cycling are not two isolated entities, but entwined.

**Response:** We thank the reviewer for highlighting this important gap in our discussion, we have expanded the discussion to emphasize that the stability of photosymbiosis is critical for providing sufficient carbon to support strobilation, and that its breakdown under stress can lead to failed strobilation. The following texts were added to our discussions:

Added Text:

The most plausible mechanistic explanation is that only a stable symbiotic relationship can provide the energetic foundation required for proper morphogenesis. The importance of stable photosymbiosis for cnidarian host growth, as well as the detrimental effects of heat stress in both adult corals and Cassiopea, are well documented: under thermal stress, algal symbionts often lose photosynthetic efficiency and retain a greater proportion of photosynthate for their own metabolism (Rädecker et al. 2021). As a consequence, the host receives insufficient translocated carbon to support development. This metabolic shortfall forces the host to shift from anabolic assimilation of carbon to catabolic consumption of endogenous reserves such as amino acids, a process linked to reductions in adult body size and mortality in Cassiopea, as well as bleaching in corals (Rädecker et al. 2021, Toullec et al. 2024). Such carbon limitation is likely to be even more acute in small-bodied ephyrae with minimal energy reserves, ultimately constraining growth and impairing proper morphogenesis.

Heat stress can alter the host-symbiont stability, potentially leading to a breakdown in mutualism. A likely driver of this paradox is the nutrient balance shift discussed above: when hosts become carbon-limited, they catabolize their own amino acids, greatly increasing ammonium (nitrogen source) availability to the symbionts, thereby fueling unchecked algal proliferation (Rädecker et al. 2021). Under such conditions, symbionts proliferate more rapidly within the host and exhibit higher dispersal rates while simultaneously reducing host growth and reproduction, and lead to increase the susceptibility of bleaching (34) (Cunning and Baker, 2013). Thermal stress has also been documented as a disruptor of host development and immune function (35), which may further exacerbate uncontrolled symbiont growth and a shift from mutualism toward parasitism.

3) It is not clear to me whether algal symbionts extracted from ephyra were normalized to anything – ephyra size, weight, protein, etc. - currently, they are only presented as cells per ml. This needs clarification.

**Response:**

We thank the reviewer for this observation. Although we measured ephyra volumes prior to flash freezing, we initially did not normalize cell counts to these volumes. The data have now been corrected and are presented as cells per ephyra volume in Figure 5b and the supplementary data. This adjustment has led us to revise the discussion and conclusions to better reflect our observations. We sincerely thank the reviewer for identifying this oversight, which has strengthene our study, and we apologize for the initial error.

4) Fig. 6 is captioned in the text, but missing.

**Response:** Fig. 6 was uploaded to the file system but not rendered into the pdf. We are sorry for the inconvenience and we made sure it was included in our revised version.

More detailed comments:

Lines 88 and following: Is there any published documentation on the clonal polyp line? I know that the polyp grow quite abundantly under culturing conditions when things are well, but it would be helpful to provide at least a citable resource (reference or protocols.io) if available, for reproducibility. Also, is the San Francisco strain of Artemia commercially available? It would be good to mention the product more specifically.

We have added the citation for the rearing protocol we conducted throughout our study (Sharp et al., 2025 - Cassiopea xamachana polyp feeding under husbandry conditions). We have added the information regarding the Artemia strain used in the methods.

Lines 109-111: I would like to suggest including Toullec et al. (2024) Microbiome (https://doi.org/10.1186/s40168-023-01738-0) here as well – this is the temperature at which bell pulsation may begin to shift into irregular spasms, and where signs of photosymbiotic breakdown in the medusa are beginning to manifest – irregularities to bell pulsation begins at 34C – in part as a justification.

**Response:** We have incorporated the reference to Toullec et al. (2024) to strengthen the justification for selecting 34 °C as the heat treatment temperature.

Revised Text: … and marks the onset of irregular pulsation and early photosymbiotic breakdown in medusae (Toullec et al., 2024).

Lines 121-123: How long did colonization take? In this paragraph, you are only referring to ‘12 h post colonization’ – is this really post colonization, or rather post inoculation? Please kindly clarify this section, and be mindful of inoculation vs colonization, which is not the same thing. Please note that in the discussion, proliferation of taken-up cells following infection, and migration (all of which I would consider to be part of the colonization process) is highlighted to start around day 8 post-infection. This needs to be clarified.

**Response:** We appreciate the reviewers' insight. We understand how the misuse of colonization is confusing. We did mean inoculation, and that has been changed throughout the paper.

Line 123-124: ‘we removed all water from the 6 well plates (…) to remove any culturing media from the symbionts’ – I would remove ‘from the symbionts’, as this can be confusing. I understand now that this is essentially the media in which the polyps were inoculated, but this can be read as if you were mistakenly referring to the polyps as symbionts. Perhaps here you could simply say ‘to remove the spent media after inoculation’?

Done.

Lines 137-138: why was host strobilation rate only counted every 3-5 days, why not e.g., daily or at every second day, and why did the time increments differ? How long does strobilation really take? My concern is that strobilation rates might be underestimated at higher temperatures if larvae are overlooked, over 5 days when no quantitation was conducted. Ephyra larvae might die and completely decompose between 3-5 days and go unnoticed.

**Response:** We thank the reviewer for raising this point and appreciate the opportunity to clarify. Although plates were checked daily, formal quantification of strobilation began 15 days post-inoculation, when the first polyps started strobilating. From day 15 to day 22, strobilation was quantified daily or every other day, as strobilation typically occurs within this period under normal conditions. During this time, plates were also checked every other day to feed and maintain the polyps. After nearly a month with no observable change in the treatment groups, we reduced quantification frequency, as changes were unlikely over a few days. The “3–5 day” intervals in the Results reflect the timing of observed new strobila in heat-stress and recovery groups. Based on these observations and daily monitoring, we are confident that ephyrae was not overlooked. We have clarified this timeline in the Methods to prevent further confusion.

Lines 143-144: was the homogenized suspension not centrifuged and washed to remove any tissue remains prior to loading the homogenate into the hemocytometer?

**Response**: The homogenized suspension was centrifuged, and the host tissue was removed, then the algal cells were resuspended in seawater and stained with Lugol prior to loading the homogenate into the hemocytometer. We have changed this in the methods by adding this information with specific details for reproducibility.

Lines 143-144: It is not clear to me whether algal symbionts extracted from ephyra were normalized to anything – ephyra size, weight, protein, etc. - currently, they are only presented as cells per ml – is this supposed to be cells per ephyra? (which is not quantitative). This needs clarification.

**Response:** We appreciate the reviewers' insight. When collecting ephyrae for freezing, we measured their diameters and calculated their volumes, but initially did not normalize the cell counts to these values. We have now corrected this, updating the supplemental data, Figure 5B, and all relevant text to present cell counts normalized to ephyra volume. The control group all had diameters of 5mm, volume of 19.63mm^3,^ the treatment group with normal morphology had diameters of 4mm, volume of 12.67 mm^3^, and the treatment group with mishapen morphology had diameters of 3mm, volume of 7.07 mm^3^. This information has been added in lines 182-184. Additionally, the total number of algal cells counted using the hemocytometer was divided by the volume of the ephyra in each sample. Cell counts were divided by the corresponding ephyra volume, and these clarification has been added, in line 191.

Lines 114-115 vs. 144-146: why were methods of algae quantitation switched between inoculation (automated cell counter) and algal counts from ephyra larvae (hemocytometer)? The two different methods have different sensitivities / error rates. Yes, it is not as problematic as counting different sets of algae cultures or different sets of ephyras with different methods, but it seems like an unnecessary switch.

**Response:** We thank the reviewer for this comment. We used a hemocytometer to count algal cells within the ephyrae because our lab’s automated cell counter cannot distinguish between ephyra cells and algal cells. To ensure accuracy, we specifically stained the algal cells with Lugol’s solution and counted them manually, thereby avoiding any confusion from counting ephyra cells.

Results and discussion: I just wanted to say that I really appreciate the combined results and discussion format. I know it’s not everybody’s cup of tea, but if the journal supports this format, I am all for it.

**Response:** We thank the reviewer for their support!

Line 168 and following: ‘symbiont cell viability’ – is viability really the best term for density of living algae? How did you discriminate between dead and live algae?

**Response:** We appreciate the reviewer’s thoughtful comments. To determine viability, we used the automated cell counter, Countess II FL, along with Trypan blue staining. When performing the cell counts, it determines the percent of the cells, as well as the total cells, that are alive or dead. When presenting our counts, we only took into account the number of living cells, which is why we presented them as viable cells. This has been clarified in line 144.

Line 170: please also provide standard errors or deviations for cell density counts – the figure suggests replication, and the point-to-point response suggests this information has been added, but it is not there.

**Response:** Done.

Lines 172-173: are there no replicates for chlorophyll content measurements – both the text and associated fig. 4b give the impression there is just one replicate for each condition and time point? The point-to-point response to one of the previous reviewers suggests ‘few measurements’, but is not clear about not having replication. I would be very careful reporting these data without replication – yes, they do make sense in the biological context, but they are not really robust.

**Response:** We thank the reviewer for this observation and agree that these data are not robust. We have added a statement clarifying that only one measurement was taken per day. While the data suggest a possible mechanism of heat-stress damage, we have revised the discussion to remove definitive conclusions, instead framing these findings as hypotheses and highlighting areas for future study.

Lines 173-174: ‘symbionts appeared lighter in color compared to dark brown algae cultures’ – this is very confusing – I only understood that two algal cultures are compared here, one in the control, versus one at heat stress when I looked at the photograph in the linked figure. I would urge the authors to be mindful of terminology – ‘symbionts’ suggested to me here that you were referring to freshly isolated symbionts from ephyra, which were contrasted to algal cultures. I would rephrase as follows:
‘Heat-stressed Symbiodinium cultures were also visibly lighter in color compared to the control cultures, supporting lower pigment content.’ Or something along these lines. While not quantitative, this would also lend tentative more support to your chlorophyll measurements, which lack replication.

**Response:** Done.

Lines 236-253: I find the discussion on the roles of photosymbiosis for ephyra development rather underdeveloped, but frankly deserving of taking up more space. Yes, the authors highlighted the effects of aposymbiosis on chemically-induced ephyra, but did not really explain why photosymbiosis is relevant. In line 253, the breakdown of mutualism is mentioned, but really brushed over, before other potential host factors are briefly mentioned. I think the role of metabolism in symbiotic nutrient cycling (which can also be a main driver of symbiont proliferation, via net release of ammonium from the starving host), and its ultimate breakdown alone deserves a few references from both the Cassiopea and coral field, e.g. Microbiome, Radecker et al. 2021 PNAS, along with the pioneering work by Ross Cunning. The breakdown of symbiotic nutrient cycling is considered the main driver of cnidarian bleaching and holobiont breakdown today. The algae fix less CO2, and/or retain more organic carbon for their own metabolism, transferring less of it to their host – which essentially starves and has to resort to burning its own proteins once it runs out of carbon. This could potentially be problematic not only for the mature host, but also for more vulnerable early developmental stages, which have very little tissue and probably not much energy storage. This can be summarized and integrated here in a sentence or two and would help make for a more well-rounded discussion.

**Response:** We thank the reviewer for pointing out this gap in our discussion and providing several highly relevant papers. We have developed more discussion on the role of healthy photosymbiosis on cnidarian development and the consequences of photosymbiosis disruption, and cited the papers.

New discussion:

While photosynthesis may not be the direct cue for host strobilation, it is essential for proper *Cassiopea* development. Aposymbiotic *Cassiopea* polyps induced to strobilate by chemical cues often exhibit developmental abnormalities, including reduced ephyra size, malformed sensory organs, crimped marginal lappets, delayed polyp recovery after strobilation, or, in some cases, death (25). Our results corroborated these findings, indicating that a healthy onset of photosymbiosis is a prerequisite for normal ephyra development. The most plausible mechanistic explanation is that only a stable symbiotic relationship can provide the energetic foundation required for proper morphogenesis. The importance of stable photosymbiosis for cnidarian host growth, as well as the detrimental effects of heat stress in both adult corals and *Cassiopea*, are well documented: under thermal stress, algal symbionts often lose photosynthetic efficiency and retain a greater proportion of photosynthate for their own metabolism (Rädecker et al. 2021). As a consequence, the host receives insufficient translocated carbon to support development. This metabolic shortfall forces the host to shift from anabolic assimilation of carbon to catabolic consumption of endogenous reserves such as amino acids, a process linked to reductions in adult body size and mortality in *Cassiopea*, as well as bleaching in corals (Rädecker et al. 2021, Toullec et al. 2024). Such carbon limitation is likely to be even more acute in small-bodied ephyrae with minimal energy reserves, ultimately constraining growth and impairing proper morphogenesis.

Why then, do ephyrae with bell abnormalities have a higher symbiont density than normal-shaped ones in the recovery group, and even the control group? Heat stress can alter the host-symbiont stability, potentially leading to a breakdown in mutualism. A likely driver of this paradox is the nutrient balance shift discussed above: when hosts become carbon-limited, they catabolize their own amino acids, greatly increasing ammonium (nitrogen source) availability to the symbionts, thereby fueling unchecked algal proliferation (Rädecker et al. 2021). Under such conditions, symbionts proliferate more rapidly within the host and exhibit higher dispersal rates while simultaneously reducing host growth and reproduction, and lead to increase the susceptibility of bleaching (34, Cunning and Baker, 2013). Thermal stress has also been documented as a disruptor of host development and immune function (35), which may further exacerbate uncontrolled symbiont growth and a shift from mutualism toward parasitism. Although we did not directly measure host fitness in this study, our findings are consistent with this interpretation. Notably, similar abnormalities of the bell, such as inversion, have been observed in *Cassiopea* raised under severe seawater acidification (pH = 7.0) (11). Further research is needed to investigate the underlying mechanisms driving these developmental abnormalities and their broader ecological implications.

Line 259: ‘inverted bell-shaped abnormalities’ is rather peculiar phrasing. I suggest rephrasing to ‘abnormalities of the bell, such as inversion’ or ‘abnormalities in shape, such as inverted bells’.

**Response:** Done

Line 284-286: Fig. 6 (asexual reproduction via budding) is captioned, but missing from the manuscript.

**Response:** Done.

Lines 288-303: this is an interesting discussion, but I find it rather one-sided. I would think that carbon limitation all by itself is a pretty strong argument as well – the algal symbionts provide energy needed for strobilation, if only in the form of ‘junk food’. The ‘checkpoint’ here being: do I even have the energy for strobilation?

Line 341 and following: I would clearly state the absence of replication for chlorophyll a content measurements here as well.

**Response:** We have added this information in line 625.

Figures:
Figure 3: ‘strobilated rate’ is unusual; suggestion to replace with ‘strobilation rate’

**Response:** We agree with the reviewer, this has been changed, and we have added the changes in the new fig3.

Figure 4 a: ‘alive algal cell density’ is unusual; suggestion to replace with ‘algal cell densities’ (as in Figure 5b) or ‘densities of viable algal cells’ (IF viability was really assessed) –

**Response:** We have changed this, and we have added as in the new fig4.

Figure 5b: I am not sure I understand the y axis here – why are is there a specific cell density explicitly mentioned in the label of y axis, and what does ‘1, 2, 3’ on the axis then represent? Does this represent 100,000, 200,000, and 300,000 cells? I suggest making the axis label more clear

**Response:** We have incoroporated these changes, thanks to the reviewer for noticing this, we have addressed thee at the new fig5.
